# SpeciesGeoCoder: Fast Categorization of Species Occurrences for Analyses of Biodiversity, Biogeography, Ecology, and Evolution

**DOI:** 10.1093/sysbio/syw064

**Published:** 2016-08-02

**Authors:** Mats Töpel, Alexander Zizka, Maria Fernanda Calió, Ruud Scharn, Daniele Silvestro, Alexandre Antonelli

**Affiliations:** 1 *Department of Marine Sciences, University of Gothenburg, PO Box 460, SE-405 30 Göteborg, Sweden*; 2 *Bioinformatics Infrastructure for Life Sciences*; 3 *Department of Biological and Environmental Sciences, University of Gothenburg, PO Box 461, SE-405 30 Göteborg, Sweden*; 4 *Universidade de São Paulo, Instituto de Biociências, Departamento de Botânica, Rua do Matão, 277, Cidade Universitária, CEP: 05508-090, São Paulo, SP, Brazil*; 5 *Universidade Estadual de Campinas, Instituto de Biologia, Departamento de Biologia Vegetal, Rua Monteiro Lobato, 255, Cidade Universitária, CEP: 13083-862, Campinas, SP, Brazil*; 6 *Gothenburg Botanical Garden, Carl Skottsbergs gata 22A, SE-41319 Göteborg, Sweden*

**Keywords:** ancestral area reconstruction, biodiversity patterns, ecology, evolution, point in polygon, species distribution data

## Abstract

Understanding the patterns and processes underlying the uneven distribution of biodiversity across space constitutes a major scientific challenge in systematic biology and biogeography, which largely relies on effectively mapping and making sense of rapidly increasing species occurrence data. There is thus an urgent need for making the process of coding species into spatial units faster, automated, transparent, and reproducible. Here we present SpeciesGeoCoder, an open-source software package written in Python and R, that allows for easy coding of species into user-defined operational units. These units may be of any size and be purely spatial (i.e., polygons) such as countries and states, conservation areas, biomes, islands, biodiversity hotspots, and areas of endemism, but may also include elevation ranges. This flexibility allows scoring species into complex categories, such as those encountered in topographically and ecologically heterogeneous landscapes. In addition, SpeciesGeoCoder can be used to facilitate sorting and cleaning of occurrence data obtained from online databases, and for testing the impact of incorrect identification of specimens on the spatial coding of species. The various outputs of SpeciesGeoCoder include quantitative biodiversity statistics, global and local distribution maps, and files that can be used directly in many phylogeny-based applications for ancestral range reconstruction, investigations of biome evolution, and other comparative methods. Our simulations indicate that even datasets containing hundreds of millions of records can be analyzed in relatively short time using a standard computer. We exemplify the use of SpeciesGeoCoder by inferring the historical dispersal of birds across the Isthmus of Panama, showing that lowland species crossed the Isthmus about twice as frequently as montane species with a marked increase in the number of dispersals during the last 10 million years.

Species distributions provide the basic knowledge for biodiversity research (Hortal et al. 2015), including our understanding of species’ environmental requirements, biogeographic history, and expected resilience to climate change. However, analyzing the distribution of the world’s estimated 8.7 million species (Mora et al. 2011) remains a major scientific challenge.

There are now approximately 644 million species occurrences available through the Global Biodiversity Information Facility (GBIF; http://www.gbif.org; accessed on April 21, 2016) and other biodiversity information networks, of which about 567 million are geo-referenced (provided with latitude and longitude data). These numbers include not only living species but also fossil taxa, allowing for biogeographic analyses based on both sources of data (e.g., Antonelli et al. 2015; Silvestro et al. 2016). Species occurrence data are steadily increasing thanks to new agreements on data sharing, on-going digitalization programs, and tools that enable automated geo-referencing of older museum specimens (Guralnick et al. 2006; Garcia-Milagros and Funk 2010).

Publicly available species occurrences represent an enormous data source for biodiversity research, but are as yet poorly exploited due to two main factors: (i) general skepticism concerning the quality of records available, in terms of species identification and precise coordinates (Robertson et al. 2016; Antonelli in press); and (ii) demonstrated taxonomic, geographic, and temporal biases (Boakes et al. 2010; Meyer et al. 2015). Improving quality elies on data curation—practiced by some country-level projects such as Flora Hellenica (Strid 2000)—as well as tools for automated data cleaning, for example through workflows such as the Biodiversity Virtual e-Laboratory (Vicario et al. 2011) and packages such as “biogeo” (Robertson et al. 2016). Finally, taxonomic misidentifications may be common (Goodwin et al. 2015), which may lead to erroneous inferences on the total distribution of a species. While an empirical assessment of the accuracy of each observation cannot be easily automated, it should be possible to assess the influence of random identification errors on the geographic ranges of species.

To make sense of biological distributions, raw species occurrences often need to be classified into discrete categories. These can then be used in connection with a phylogeny for historical biogeographic analyses including ancestral range reconstructions (Ree and Smith 2008; Landis et al. 2013; Matzke 2013) and area-dependent inferences of diversification rates (Silvestro et al. 2011; FitzJohn 2012). Species categorization into commonly recognized areas such as eco-regions and realms (Olson et al. 2001; Abell et al. 2008; Holt et al. 2013) and biogeographic regions or bioregions (Vilhena and Antonelli 2015; Edler et al. this issue) may reveal patterns of biodiversity and distribution at a large scale, facilitating the identification of regions with outstanding levels of species richness and endemism that is central to the concept of biodiversity hotspots (Myers et al. 2000). Rapidly increasing species occurrence data and the need to classify species into discrete areas in an automated, reproducible, and transparent way has led us to develop SpeciesGeoCoder.

## Description

**Figure 1.**
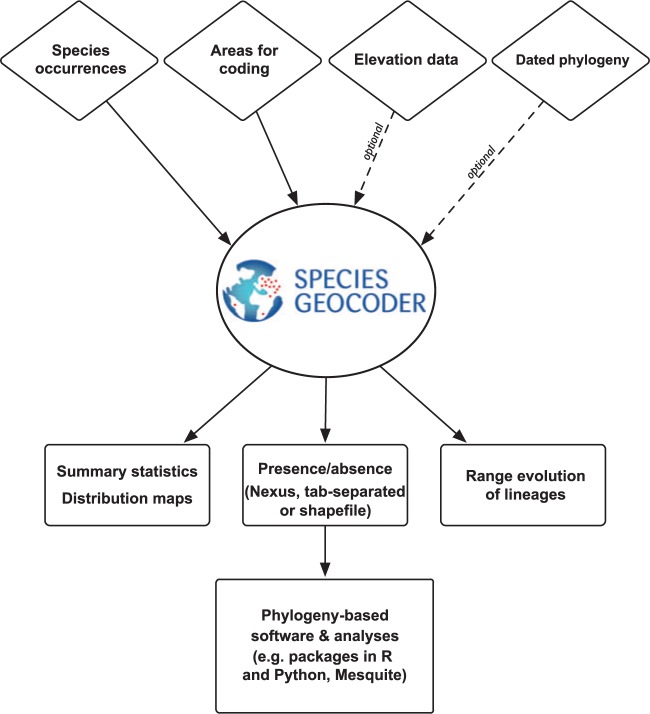
Simplified workflow of the SpeciesGeoCoder package. See text for details.

The source code consists of a set of python modules, centered on a point-in-polygon algorithm that determines if a species locality record is found inside or outside of a particular polygon. The analysis of geoTIFF files is done using the GDAL python bindings (http://www.gdal.org/) for fast execution. The basic workflow is illustrated in Figure 1 and described below:

1. The user provides two files. The first should contain species occurrence data, including species names, latitude, and longitude in either (i) tab-separated format, (ii) the CSV format provided by www.gbif.org, or (iii) in shapefile format. The second file defines the areas of interest in tab-separated format (i.e., a list of named polygons, and—optionally—an elevation or sea depth range such as between 1000 and 2000 m a.s.l., or between 0 and $-$30 m) or as a shapefile. Polygon files can be generated easily with various GIS tools, and a tutorial is available at https://github.com/mtop/speciesgeocoder/wiki for the freely available program QGIS (http://qgis.osgeo.org). If an elevation range is provided for the polygons, then elevation data in the form of geotiff files is also required, and these can be downloaded from various online resources (see the wiki for tutorials, links, and example files);
2. SpeciesGeoCoder loops through the input file and counts the number of occurrences of each species in each polygon, and optionally takes the elevation constraints into account;
3. The default output format is a Nexus file containing a data matrix with all analyzed species and their presence (1) or absence (0) in each area. “Presence” requires by default at least one occurrence in an area, but user-defined thresholds may be set instead. This means that outlier localities incorrectly assigned to a polygon (e.g., due to an erroneous shift between latitude and longitude) can be easily identified. Alternatively, the user may request a more complex output including the number of occurrences in each polygon, which could further aid the identification of outliers. The Nexus file can then be analyzed in programs such as Mesquite (Maddison and Maddison 2009) and most phylogenetic packages written in R, such as APE (Paradis et al. 2004), geiger (Harmon et al. 2008), Diversitree (FitzJohn 2012), BayArea (Landis et al. 2013), and BioGeoBEARS (Matzke 2014), as well as others written in Python, such as BayesRate (Silvestro et al. 2011), and Biopython (Cock et al. 2009). SpeciesGeoCoder can also export the result of an analysis in tab-separated text format for easy parsing and additional analyses. Localities identified inside any of the polygons analyzed can also be exported in shapefile format, which gives a convenient way of extracting a subset of localities from a larger data set;
4. The second (optional) type of output is a series of summary statistics and distribution maps. These include multiple pdf documents with bar charts as graphical representations of the number of species per area, the number of occurrences per species per area, and the relative occurrence per area for each species. The summary tables used for the graphical output are also made available as tab-separated text files. The distribution maps plot all occurrence points and the areas included in the analyses. In addition, for small data sets comprising less than 40 species, a coexistence matrix for each area is calculated and visualized as a heat map. These files help not only with biological interpretations, but also to identify problematic occurrence points that need further verification;
5. The third (optional) output is a series of plots summarizing the historical dispersal of lineages between all pairs of user-defined areas, based on one or a sample of dated phylogenies. These plots are generated with R scripts, using by default stochastic mapping to infer shifts in transitions along branches, and the computation of absolute as well as relative (i.e., corrected by the number of lineages) number of dispersals through time (Silvestro 2012; Fernández-Mendoza and Printzen 2013; Antonelli et al. 2015);
6. The fourth (optional) output includes a sensitivity test aimed at quantifying the potential effects of incorrectly identified specimens in the input data on the coded geographic ranges. In this test, we assume that each geo-referenced occurrence has a probability $r$ to be misidentified. An occurrence mistakenly assigned to a species should be removed from the data when coding the species distribution. However, its removal may or may not change the geographic distribution of the species coded within discrete areas depending on whether other (correctly identified) occurrences of the species are found in the same geographic unit. In our sensitivity test, occurrences that are randomly selected as misidentified are removed from the data and the geographic distribution of the species is re-coded based on the sub-sampled occurrences. This procedure is repeated for all occurrences 10,000 times under error probabilities equal to 0.05, 0.10, 0.25, and 0.50. The results of these tests are summarized in terms of average number of species in a data set that change in their coded range under different scenarios of errors, compared to their ranges estimated from all occurrence data. Additionally, we compute the probability that a random error affects the coded distribution of each species and provide these probabilities for each species in a data set (thereby identifying which species are most sensitive to error given the number of occurrences and their distribution under different error probabilities). These results are saved in tab-separated tables.

The overall design of SpeciesGeoCoder is done with extensibility in mind. The package is composed of a set of python classes for storing and manipulating locality and polygon data in different input formats, so users with experience in object-oriented programing should be able to extend the package with additional features or contact the authors for proposing novel implementations.

## Benchmark

We tested the performance and scalability of our package through a series of simulations on a five-year-old computer with four 12-core 1.9 MHz AMD Opteron 6168 processors. All benchmarks, except the ones examining multiprocessor performance, were ran on one CPU and we focused on how computing time was determined by three key variables: (i)the number of geo-referenced occurrences, (ii) the number of polygons, and (iii) the complexity of the polygons, measured by their number of corners (i.e., vertices or nodes). An occurrence dataset (i) was simulated as a set of globally distributed localities (see example in Supplementary Fig. S1 in Supplementary Material). The polygon dataset (ii) was generated in a similar way, by creating a grid of square polygons sharing two corners with each of its neighboring polygons (Supplementary Fig. S2 in Supplementary Material). The polygon complexity dataset (iii) was generated by creating one square polygon, and successively adding nodes equally distributed over its perimeter (Supplementary Fig. S3 in Supplementary Material). This approach of generating polygons with an increasing number of coordinate pairs (i.e., nodes) is suitable for benchmarking purposes since the computation time for the point-in-polygon algorithm implemented in SpeciesGeoCoder is not affected by the actual shape of the polygon, but only by the number of coordinates that make up the polygon. The simulations were then performed with a logarithmical increase in the number of occurrences, polygons, and polygon nodes, ranging between 10$^{1}$ and 10$^{8}$.

**Figure 2.**
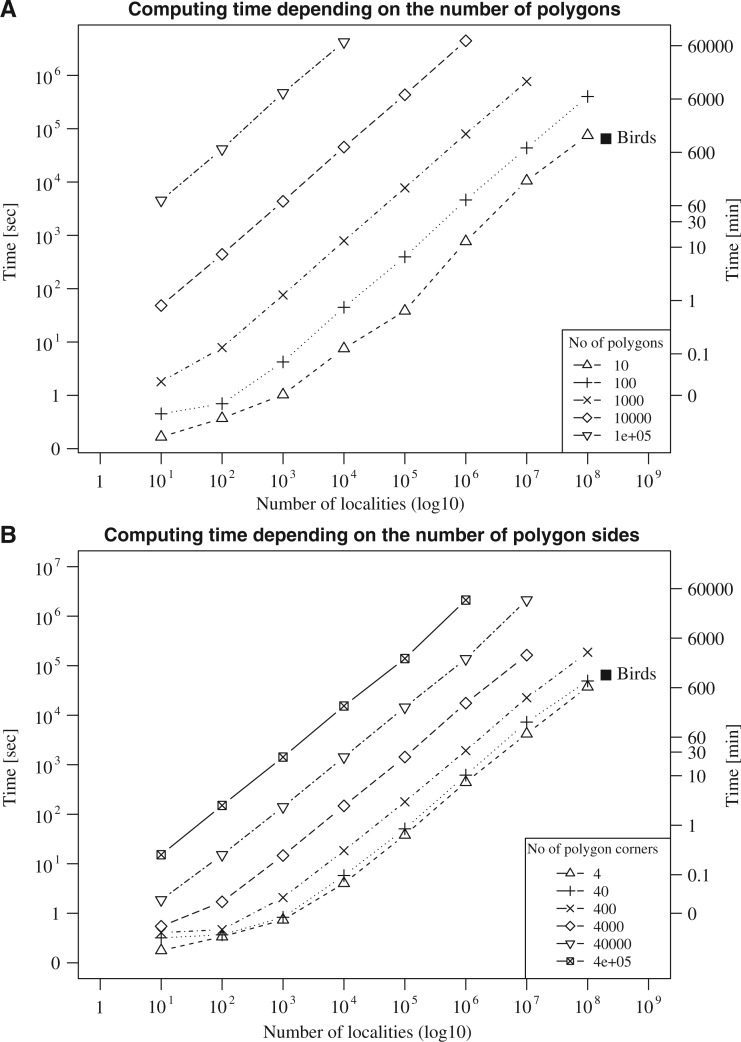
Computational time in relation to the increase in A) number of polygons and B) polygon complexity (number of polygon corners) and number of species occurrences. As a comparison to empirical data, the square labeled “Birds” corresponds to the coding of approximately 200,000,000 bird occurrences available from ebird.org. The analyses were performed on a standard computer with a 1.9 MHz processor.

Our results (Fig. 2) show that there is a nearly linear relationship between the computation time required and (i) the number of localities, (ii) the number of polygons, and (iii) the complexity of the polygons analyzed. Doubling the amount of input data will also double the analysis time. In addition, we examined how well the parallelization of the code worked by running analyses on 10 million localities and 100 polygons using 1, 2, 4, 8, 16 and 32 CPUs. We found a negative linear correlation between the computation time and the number of CPUs utilized, and that doubling the number of CPUs will decrease the computation time with nearly 50%. These results lead us to the conclusion that SpeciesGeoCoder can handle vast amounts of data—millions of polygons and occurrences—within feasible time using standard computers.

## Biological Example

**Figure 3.**
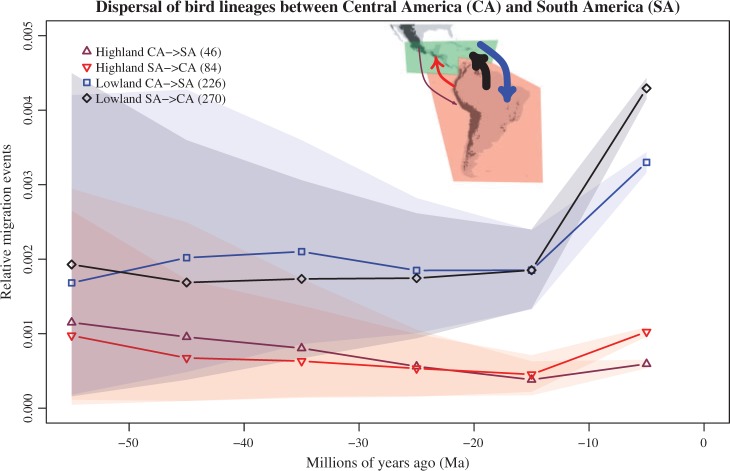
Historical dispersal of lowland and highland bird lineages between South America (SA) and Central America (CA), calculated per 10-million-year time bins. The total number of dispersal events inferred for our data set is indicated between brackets in the legend. The inset shows the two polygons used for coding all bird species, with arrows colored as in the curves for migration events and their width proportional to the total number of events.

We inferred the historical dispersal of montane and lowland bird lineages through time across the Central American Seaway, which separated North and South America for millions of years until the emergence of the Isthmus of Panama (Bacon et al. 2015a, 2015b; Montes et al. 2015). First, we downloaded the full occurrence data set for all birds including approximately 10,000 species and 200,000,000 records from http://www.ebird.org (eBird 2013). We then used SpeciesGeoCoder to exclude all records found outside the South American continent, as well as all those found north of the Tropic of Cancer, and coded the remaining species into Central America and South America. We defined the border between South and Central America following the Uramita fault (Montes et al. 2012) that separates the South American and the Panamanian geological plates (Fig. 3). We created two operational units from each polygon, one including occurrences below 1000 m a.s.l. (lowlands) and the second including occurrences above 1000 m a.s.l. (highlands) following the same categorization as Weir (2006). We then reconstructed ancestral areas onto the species-level dated phylogeny of birds provided by Jetz et al. (2012), using stochastic mapping to reconstruct the historical dispersal of lineages through time among these four operational units. We calculated both the total (absolute) as well as the relative (in proportion to the number of lineages) number of dispersals between each pair of areas, using bins of 10 million years. Since not all bird species could be matched between the phylogeny and the occurrence data set, the final analyses included 4350 species.

We tested the sensitivity of the geographic categorization of bird species to identification errors using the procedure described above. A random error equal to 5% would affect the coded geographic distribution of 48 ($\pm 11$) species (${\sim}1{\%}$ of the dataset), whereas a 10% error frequency would affect 79 ($\pm 14$) species (${\sim}2{\%}$). Only a very high error frequency of 50% (where one in two samples would be wrongly identified to species level) would affect more than 5% of the species in our dataset ($250 \pm 25$ species), thus indicating that area categorization in this case is robust to random identification errors. Detailed output of the sensitivity test is provided in Supplementary Table S1 in Supplementary Material (available at Dryad at http://dx.doi.org/10.5061/dryad.tm32k).

Although the variation among reconstructions is large, our results suggest that dispersals between the lowlands of South and Central America occurred consistently more frequently (c. 2–4 times) than dispersals between the highlands of those landmasses (Supplementary Figures S3 and S4 in Supplementary Material). There were no major differences in directionality of dispersals, except for the last time bin considered (0–10 Ma) when northward dispersals dominated. This supports the conclusion by Weir et al. (2009) that birds mainly followed an opposite route during the Great American Biotic Interchange as compared with mammals, which migrated mostly southwards and in more recent times (Stehli and Webb 1985; Carrillo et al. 2014; Bacon et al. 2016). The rate of dispersals increased for all categories in the last time bin, probably as a consequence of the emergence of the Panama Isthmus in the last 13 Ma (Bacon et al. 2015a, 2015b; Montes et al. 2015). Dispersals in previous time periods seem to follow a rather uniform, stochastic rate (de Baets et al. in press) that is also reflected by sporadic fossil findings such as a recently described South American primate in the early Miocene (20.9 Ma) of Panama (Bloch et al. 2016).

## Conclusions

We have shown that SpeciesGeoCoder allows for easy and fast categorization of species distribution data for various analyses in biogeography, ecology, and evolution. Beyond the example provided here, the output obtained could be readily used for calculating measures of alpha, beta, and gamma diversity; the identification of neglected areas for conservation; and providing real-time detection of GPS-tagged animals entering and leaving protected areas. Finally, the visualization and coding of species into areas may greatly facilitate cleaning up occurrence databases, by enabling the identification of outliers that may require additional examination or exclusion from subsequent analyses (Maldonado et al. 2015).

Although several of the functions in SpeciesGeoCoder could be performed in other software by an advanced GIS user, our package offers a number of advantages: it is fast, increases reproducibility of analyses, allows exploration of alternative sets of polygons for area coding, can handle large files, is particularly suitable for phylogenetic and biogeographic analyses, enables the inclusion of thresholds for coding, assesses the effect of misidentified specimens, includes elevation or depth, allows for batch processing, is directly integrated with stochastic mapping, produces summary tables and maps, among others. We therefore hope that SpeciesGeoCoder will become an indispensible tool for coding species into discrete units, as is required for most currently available parametric methods in historical biogeography and for the exploration of biodiversity patterns.

## Availability

SpeciesGeoCoder is available for download from https://github.com/mtop/speciesgeocoder/releases. The current release includes installation instructions for Mac OSX, Gnu/Linux, and Windows; example files, tutorials, plug-in scripts, and useful links. The program with complementary functions is also available for the R environment (speciesgeocodeR) from CRAN at http://cran.r-project.org/web/packages/speciesgeocodeR/index.html, as well as via a web interface at http://portal.bils.se/speciesgeocoder/tool. Links to all resources are available at https://github.com/mtop/speciesgeocoder/wiki, https://github.com/azizka/speciesgeocodeR/wiki or from http://antonelli-lab.net.

## Supplementary Material

Supplementary material, including data files and online-only appendices, can be found in the Dryad data repository at http://dx.doi.org/10.5061/dryad.tm32k.

## Funding

Funding was provided by the Swedish Research Council (B0569601), the European Research Council under the European Union’s Seventh Framework Programme (FP/2007-2013, ERC Grant Agreement no. 331024), and a Wallenberg Academy Fellowship to A.A.; from Carl Tryggers stiftelse (CTS 11:479, CTS 12:507) to M.T.; and from Fundação de Amparo à Pesquisa do Estado de São Paulo (FAPESP 2009/52161-2, 2013/10262-2) to M.F.C.
